# Lithotomy and Calculous Diseases

**Published:** 1853-04

**Authors:** 


*Lithotomy and Calculous Diseases.* From the Report of Prof. Gross to the Kentucky Medical Society.—In no department of Surgery has Kentucky acquired more renown than in the treatment of calculous diseases. The operations that have been performed for the removal of stone from the bladder, nearly equal, if, indeed, they do not exceed, all the operations of lithotomy in the other States of the Union. The gentleman—Professor Dudley, of Lexington—who stands at the head of this department of Surgery, not only in Kentucky, but in America, if, in fact, not in the world, has alone operated two hundred and seven times; and, if we add to these cases those that have occurred in the hands of other surgeons, the aggregate will amount to nearly, if not quite, four hundred. It would afford me great pleasure to give, with exact accuracy, the number of operations of lithotomy that have been performed by different members of the profession in different parts of the State, as well as their results; but, for the determination of this question, so interesting and important to the profession, I have not, I regret to say, been able to obtain the requisite data, notwithstanding that no pains have been spared to do so.

It is positively known that the late Dr. E. McDowell, of Danville, had operated thirty-two times several years before he died. Dr. Alban G. Smith, now Dr. Goldsmith, *thinks* that, during his residence in Kentucky, he had over fifty cases.* Dr. Gardner, of Woodsonville, has reported to me fourteen cases. Dr. Bush, of Lexington, has operated six times; Dr. John C. Richardson, and Dr. E. L. Dudley, of the same city, each once. Dr. John Shakleford, of Maysville, has had four cases; and Dr. Craig, of Stanford, two. Dr. W. A. McDowell, during his residence at Danville, operated twice. Dr. Henry Miller, of this city, has had two cases. Dr. John Hardin, while practicing medicine and surgery at Greensburg, operated five times; Dr. L. P. Yandall has operated four times; Dr. William H. Donne, once; Dr. Samuel B. Richardson, twice; and Dr. Joshua B. Flint, .. times.† My own cases amount to thirty. Dr. Walter Brashear, formerly of Bardstown, occasionally operated for stone in the bladder, but how frequently I am unable to say. I am in the possession of a few of the particulars of one of his cases, which I shall take occasion to relate in their proper place. Dr. D. W. Yandell, late of this city, has performed lithotomy four times.

Reduced to figures, the number of cases of lithotomy positively known to have occurred in Kentucky, stand about thus:

| | |
|---|---|
| Dr. B. W. Dudley, | 207 |
| Ephraim McDowell | 32 |
| A. Goldsmith, uncertain | 50 |
| W. Gardner | 14 |

* Letter to the Reporter.

† This gentleman has not honored me with an account of his cases.

| | |
|---|---|
| Dr. J. M. Bush | 6 |
| John Shackleford | 4 |
| Henry Miller | 2 |
| John Hardin | 5 |
| Samuel B. Richardson | 2 |
| John C. Richardson | 1 |
| John Craig | 2 |
| William H. Donne | 1 |
| Walter Brasbear | .. |
| E. L. Dudley | 1 |
| D. W. Yandell | 4 |
| L. P. Yandell | 4 |
| S. D. Gross | 30 |

It is an interesting fact, with reference to the etiology of calculous affections, that most of the above cases occurred among the inhabitants of Kentucky and Tennesse. The remainder were brought from Alabama, Mississippi, Indiana, Illinois, Missouri, Ohio and Virginia; a few, perhaps, from other States. Nearly all occurred in white males, the number of blacks being, comparatively, very small. The great majority of the patients resided in limestone regions.

The following facts and cases, illustrative of the operation of lithotomy in Kentucky, have been kindly communicated to me by different gentlemen, during the progress of my labors. It is a source of regret to me that none of the particulars of the late Dr. Ephraim McDowell's operations have been transmitted to me.

I have it in my power, through the politeness of Dr. S. M. Bemiss, of Bloomfield, to present you with a brief occount of one of Dr. Brashear's cases, accompanied with the calculus. The details are, unfortunately, imperfect.

The operation was performed in the summer of 1811, in the neighborhood of Bloomfield, and in the presence of Dr. Harrison, of Bardstown, Dr. John Bemiss, and Dr. Merrifield, the latter of whom is the only surviving witness. The subject, Mr. Falkerson, near sixty years of age, had suffered so much for some months previous to the operation, that his constitution was greatly impaired in consequence. Strong doubts seem to have been entertained by Dr. Brashear respecting his ability to endure an operation of such magnitude; but his patient was tired of suffering, and wished to be relieved by death, or a successful removal of his calculus. Dr. Merrifield thinks that the operation was performed on the left side of the perineum, with the gorget. After the proper incisions were made, the forceps were introduced, and an attempt made to grasp the stone, but the blades of the instrument could not be expanded sufficiently to admit it. The patient soon becoming exhausted, the operator was obliged to desist from any protracted efforts at extraction, hoping, perhaps, that he might be able to renew them shortly afterwards. In this, however, he was disappointed, for Falkerson died during the ensuing night, and Dr. Merrifield removed the calculus, which I here exhibit to you, after his death. Its weight was originally considerably over seven ounces, but by attrition, drilling, and the evaporation of its fluid constituents, it has been reduced so much as to amount at present only to a little more than four ounces. It is of an oblong, ovoidal shape, rough on the surface and laminated in its structure, but what its composition is I know not. Its short circumference is six inches; the long seven and a half.

Dr. Merrifield speaks in high terms of the skill and celerity with which Dr. Brashear accomplished his operation, and thinks that the debility and exhaustion of the patient were the only obstacles to the successful removal of the calculus.

In 1808 Dr. Brashear performed the operation of lithotomy upon a boy, about twelve years of age. The case, which presented nothing unusal, was perfectly successful.*

Dr, William A. McDowell, during his residence at Danville, many years ago, operated twice for stone in the bladder. The first case, which was remarkable on account of the early union of the wound, was that of a youth of eighteen, cut with the gorget. No urine passed through the perineum after the operation, the parts having closed by the first intention. The recovery was rapid and uninterrupted.

In the other case, the patient, a lady, aged thirty three, suffered great torments from the calculous affection, in

* MS. letter of R. B. Brashear, Esq., to the Reporter.

addition to incontinence of urine. In March, 1820, four stones, about the size of walnuts, were extracted from the bladder by the lateral operation. The wound healed without difficulty, and with relief from all anoyances, save the incontinence of urine, which continued up to the time of Dr. McDowell's removal to Virginia, three months afterwards.

In February, 1826, Dr. John C. Richardson, of Lexington, performed the lateral operation of lithotomy upon a youth of twenty-one, the only instruments used being a scalpel and a staff grooved on its left side. The stone was of large size, and could not be extracted without the dieision of both sides of the prostate, in the manner since so strenuously recommended by the late Mr. Liston. It was of the ammoniaco-magnesian phosphate variety, five ounces and a fourth, and was three inches and a half in its long, by two inches and a quarter in its short, diameter. The case, as I am infomred by Dr. Samuel B. Richardson, of this city, a brother of the operator, was perfectly successful.

Dr. Henry Miller, in 1830, during his residence at Harrodsburg, operated upon a mulatto boy, aged three years and a half, for stone in the bladder. He belonged to Capt. Wm. Robinson, of Anderson county, Kentucky, and had suffered from symptoms of calculus since very early infancy. The stone was quite friable, and broke into a number of fragments in the extraction. The boy recovered, and had no return of the disease during the two or three years he was under Dr Miller's observation.

In August, 1849, Dr. Miller operated upon a young girl, in her fifthenth year, from Meade county, Kentucky. After having given chloroform, he introduced a pair of common curved polypus forceps into the bladder, seized and extracted a very rough stone, resembling a Jamestown bur, weighing 264 grains, and measuring a little over an inch in diameter. The lacerated mucous lining of the urethra hung out at the meatus, after the operation, and was clipped off with the scissors. She had no bad symptoms, and went home in a few days. She has remained well ever since.

Dr. John Shackleford, of Maysville, writes that he has operated four times for stone in the bladder, and in every instance successfully. One of his patients, who became afterwards intemperate, suffers occasionally from incontinence of urine.

Dr. S. B. Richardson, of this city, during his residence at Lexington, operated twice for stone in the bladder. In one of the cases he removed three calculi, two of which were so large as almost to fill the organ. The patient, three and a half years old, had a speedy recovery, although she labored, at the time, under serious disease of the rectum and bladder.

Dr. L. P. Yandell has operated for stone in the bladder four times, using the gorget in every instance. The first three cases were successful; the fourth, a little boy, four years old, in bad health, died, never having fully recovered from the shock of the operation.

Dr. D. W. Yandell has performed the lateral operation of lithotomy four times; in all with the bistory, according to Liston's method, and in all with success. One of the subjects was a man, aged twenty years, who had suffered long with the stone; the other three were boys, in their fourth year. The recovery in each case was prompt and perfect. The patients all resided in Tennessee.

Dr. Gardner, of Woodsonville, has performed the operation of lithotomy fourteen times; using the knife and adopting the lateral method. All his patients, save one, recovered. Death in this case was produced, as was supposed, by the accidental fracture of the calculus, in the attempt at extraction, thereby necessitating its removal piece-meal, a work of great pain, difficulty and danger. Acute cystitis immediately supervened, and resisting every effort to arrest it, terminated fatally on the fourth day after the operation. The patient was a man aged twenty-five.

Of the above cases, four were from Barren county, two from Warren, two from Green, one from Taylor, one from Edmonson, one from Hardin, and three from Hart, the residence of the operator. In all these counties the limestone formation prevails, and limestone water is universally used for drinking and cooking purposes.

All the patients were males, and all, save one, were white. One was a blacksmith, and one the son of a merchant; all the rest, except one, were farmers or the children of farmers. Two of the patients were brothers. There was not a single instance of double or multiple calculus, and only one case in which a stone formed after the operation.

The ages varied from five to forty-two, as follows: 5, 7, 7, 7, 7, 7, 9, 10½, 16, 17, 19, 20, 25, 42.

In one of his cases, Dr. Gardner, after having, in vain, endeavored to extract the stone by the lateral operation, was obliged to open the bladder above the pubes. The patient was a man, aged forty-two years, for the last fifteen of which he had suffered under symptoms of the disease. Having incised the perineum, the operator was absolutely unable even to introduce the forceps into the bladder, much less to expand them over the concretion, the weight of which proved to be nine ounces, while it measured nine inches and a quarter in its greater circumference, by three and a half in diameter. Undaunted by so unexpected an occurrence, Dr. Gardner at once determined to perform the supra-pubic operation, and after some difficulty, owing chiefly to the large bulk of the stone, and the firm contraction of the bladder, succeeded in accomplishing his object. The patient recovered slowly, but without much trouble, and by the eighteenth day was able to walk about his room. The upper wound soon healed, but a small fistulous aperature remained in the perineum for some months, when it finally closed. Two years and a half after the operation, upon sounding the man, Dr. Gardner found that the bladder again contained a large calculus. A short time after this he was seized with an attack of intermittent fever, which, added to his other afflictions, soon carried him off. The body was not examined.

